# The EuRRECa Project as a Model for Data Access and Governance Policies for Rare Disease Registries That Collect Clinical Outcomes

**DOI:** 10.3390/ijerph17238743

**Published:** 2020-11-25

**Authors:** Salma R. Ali, Jillian Bryce, Li En Tan, Olaf Hiort, Alberto M. Pereira, Erica L. T. van den Akker, Natasha M. Appelman-Dijkstra, Jerome Bertherat, Martine Cools, Olaf M. Dekkers, Yllka Kodra, Luca Persani, Arelene Smyth, Christopher Smythe, Domenica Taruscio, S. Faisal Ahmed

**Affiliations:** 1 Developmental Endocrinology Research Group, Royal Hospital for Children, University of Glasgow, Glasgow G51 4TF, UK; salma.ali@glasgow.ac.uk (S.R.A.); charmainetan.lien@gmail.com (L.E.T.); 2 Office for Rare Conditions, University of Glasgow, Glasgow G51 4TF, UK; jillian.bryce@glasgow.ac.uk (J.B.); pag@officeforrareconditions.org (A.S.); Chris.Smythe@glasgow.ac.uk (C.S.); 3 Department of Paediatrics and Adolescent Medicine, Division of Paediatric Endocrinology and Diabetes, University of Lübeck, 23562 Lübeck, Germany; Olaf.Hiort@uksh.de; 4 Department of Medicine, Division of Endocrinology, Leiden University Medical Center, 2333 ZA Leiden, The Netherlands; A.M.Pereira@lumc.nl (A.M.P.); N.M.Appelman-Dijkstra@lumc.nl (N.M.A.-D.); o.m.dekkers@lumc.nl (O.M.D.); 5 Department of Pediatrics, Division of Pediatric Endocrinology, Erasmus MC-Sophia Children’s Hospital, 3000 CB Rotterdam, The Netherlands; e.l.t.vandenakker@erasmusmc.nl; 6 Obesity Center CGG, Erasmus MC-Sophia Children’s Hospital, 3000 CB Rotterdam, The Netherlands; 7 Reference Center for Rare Adrenal Disorders, Cochin Hospital, Université de Paris, 75006 Paris, France; jerome.bertherat@aphp.fr; 8 Department of Internal Medicine and Paediatrics, Ghent University, 9000 Ghent, Belgium; Martine.Cools@uzgent.be; 9 Department of Paediatric Endocrinology, Ghent University Hospital, 9000 Ghent, Belgium; 10 Departments of Medicine & Clinical Epidemiology, Leiden University Medical Centre, 2300 RC Leiden, The Netherlands; 11 National Centre for Rare Diseases, Istituto Superiore di Sanità, 00161 Rome, Italy; yllka.kodra@iss.it (Y.K.); domenica.taruscio@iss.it (D.T.); 12 Division of Endocrine and Metabolic Diseases, Istituto Auxologico Italiano, 20145 Milan, Italy; luca.persani@unimi.it; 13 Department of Biotechnology and Translational Medicine, University of Milan, 20133 Milan, Italy

**Keywords:** registries, databases, European reference networks, endocrinology, rare diseases, rare conditions

## Abstract

Rare disease (RD) registries are important platforms that facilitate communication between health care professionals, patients and other members of the multidisciplinary team. RD registries enable data sharing and promotion of research and audits, often in an international setting, with the overall aim of improving patient care. RD registries also have a fundamental role in supporting the work of clinical networks such as the European Reference Networks (ERNs) for rare diseases. With the recent expansion of RD registries, it has become even more essential to outline standards of good practice in relation to governance, infrastructure, documentation, training, audits and adopting the Findable, Accessible, Interoperable and Reusable (FAIR) data principles to maintain registries of high quality. For the purpose of this paper, we highlight vital aspects of data access and data governance policies for RD registries, using the European Registries for Rare Endocrine Conditions (EuRRECa) as an example of a project that aims to promote good standards of practice for improving the quality of utilization of RD registries.

## 1. Introduction

Rare diseases (RDs) are defined by the European Union (EU) as life-threatening or chronic conditions with a prevalence of less than 5 per 10,000 [1]. RD registries aim to improve patient care through sharing of data, public health surveillance, facilitating communication between members of the multidisciplinary team, including patients and promoting research and audits [2]. RD registries also have a key role in supporting European Reference Networks (ERNs) for RDs and the EU has identified a primary aim of ERNs as “reinforcing epidemiological surveillance like registries” [3]. There has been a recent expansion in the number of RD registries, with over 750 RD registries currently reported to exist within Europe, and over 80% of these are reported to be registries held in publicly funded institutions [4]. However, these registries are often diverse in nature and supported by varying data governance criteria and models. A need to develop guidance for the minimum standards necessary to maintain high-quality registries and to improve the quality of existing registries has recently been highlighted [5]. Kodra et al. reported on a list of recommendations, developed by a group of experts, including patient support groups, to be used as a framework for improving the quality of RD registries [5]. The list of recommendations includes aspects of governance including data sharing and access, infrastructure, documentation, training, audits, as well as a call to adopt the Findable, Accessible, Interoperable and Reusable (FAIR) data principles of data management.

The international registration of patients with RD is also encouraged by the EU [6] and several European initiatives such as Orphanet [7] and RD-Connect [8] have attempted to identify existing registries. The European Reference Network for Rare Endocrine Conditions (Endo-ERN) is the largest ERN and includes 86 reference centres from 25 countries. A recent study reported the results of a search of Orphanet and RD-Connect for international registries that exist within Europe and a survey amongst Endo-ERN centres. The study found that international registries existed for 76% of conditions covered within Endo-ERN and experts were aware of less than half of the registries that currently exist for rare endocrine conditions [9]. Therefore, although there is a need to develop new registries, there is a more immediate need to improve the awareness and participation in existing registries. The European Registries for Rare Endocrine Conditions (EuRRECa; eurreca.net), a new project which incorporates the development of a core registry directing users to high-quality detailed registries, has the potential to fulfil this objective.

The focus of this review will be to discuss vital aspects of data access and data governance policies for RD registries, using EuRRECa as an example of a new project that aims to promote good standards of practice that will lead to a greater likelihood of long-term sustainability.

## 2. Principles of Data Governance

**Figure 1:**
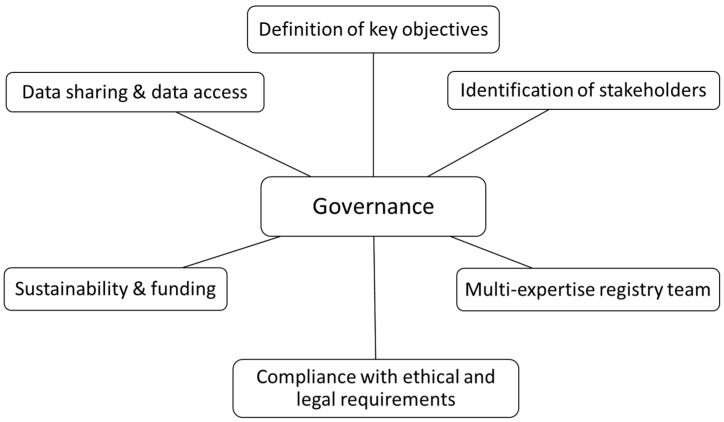
Key principles of governance of a registry.

The governance of a registry refers to guidance and high-level decision-making that is required for efficient and effective long-term operation and sustainability of the registry. Registries should have a governing structure in place and key principles include the following: definition of objectives, identification of stakeholders, a registry team and ethical, legal and societal issues (ELSIs) including privacy and ensuring sustainability (Figure 1). The structure of the governance system depends on the complexity of a registry. A registry should outline its primary and secondary objectives clearly at the outset and these objectives should be attainable as this will help to ensure that the structure and design of the database supports the collection of data that is sufficient to fulfil the objectives. The identification of key stakeholders at an early stage in the implementation of a registry is also recommended, especially the inclusion of patient representatives or support groups. Other stakeholders may include researchers, pharmaceutical companies, registry funders or private organisations. The inclusion of these stakeholders in the registry may yield several benefits including increased patient enrolment through active engagement and additional financial support.

Furthermore, the establishment of a registry team with clear roles and responsibilities designated to team members is important. The team should have differing and complementary expertise and should include a named registry lead, project manager, personnel with statistical expertise, data stewardship and database management, development and hosting and legal specialists. It is desirable that the management team is also supported by a steering committee or project governing board. With regard to ELSIs including privacy, the registry should ensure compliance with international, national and local ethical and legal requirements, and on this basis develop public policies for accessing, maintaining and operating the registry. Whilst adherence to the “General Data Protection Regulation (GDPR)” [10], applied across Europe in 2018, is essential for ensuring registry compliance across several countries within the EU, local legal requirements in several countries as well as health care centres need to be addressed. Registries should also ensure transparency, with information readily accessible to any interested parties. Another principle in the good governance of a registry is centred around ensuring sustainability. Recent studies have shown that low-quality registries are more likely to have been set up with few or no funds [11]. Thus, the registry should have policies in place to ensure that it is well-resourced for a pre-defined period and registries may seek funding from complementary sources including pharma, patient organisations, professional associations and the third sector. Registries should also have clear policies regarding data access and data sharing procedures. These policies should be transparent and accessible via a public domain (e.g., registry website) so that data can be shared with other registries at the national and international levels.

To understand the variation that may exist in the governance of endocrine registries in Europe, a questionnaire-based evaluation was recently performed of the international registries that were identified in the mapping exercise from two years ago [9]. Of the 31 registries approached, information was available for 22 registries (71%). Of these 22 registries, 21 (95%) had a registry lead and a project management group, 18 (82%) had a steering committee and an active funding stream, with 19 (86%) having documents outlining the standard operating protocol. An active web interface and user access policies were available for 21 (95%) and 20 (91%) registries, respectively. In addition, 17 registries (77%) had data access policies and data sharing agreements, 16 registries (73%) had a data access committee, patient consent forms and a registry newsletter. Twelve registries (55%) had involvement from patient organisations.

## 3. EuRRECa Project

The EuRRECa project was launched in February 2018 through funding by the EU Health Programme with additional support from the European Society for Paediatric Endocrinology (ESPE) and the European Society of Endocrinology (ESE) and is open to all expert centres. The ultimate aim of the project is to maximise the opportunity for patients, health care professionals and researchers to participate and use high-quality, patient-centred registries for a wide range of rare conditions. EuRRECa consists of two centralised registries: a platform that allows electronic reporting of new clinical encounters (e-Reporting of Rare Conditions—e-REC) and another platform that acts as a Core Registry, collecting a common dataset for several rare conditions covered within Endo-ERN (https://endo-ern.eu/) and European Reference Network on Rare Bone Diseases (ERN-BOND; http://ernbond.eu/). These registries comply with EU GDPR [10] and are approved by the Information Governance authorities at the NHS Greater Glasgow and Clyde Health Board and the National Research Ethics Service in the UK. The project receives guidance from expert working groups that align with the condition-specific groups of Endo-ERN. This guidance flows through work packages that review the needs of patients and parents, comply with the highest ethical standards and maintain high levels of quality and interoperability [5]. As mentioned in the article by Kodra et al. [5], quality and interoperability are considered to be two of the most important elements in the establishment and maintenance of the registry. High-quality data are the basis for generating knowledge upon which decisions are made and interoperability should be based on FAIR principles (i.e., Findable, Accessible, Interoperable and Reusable) in order to make data available for wider use. It is also important to collect and implement patient reported outcome (PRO) tools into the registry, such as such as EQ5D and PROMIS, that have been extensively validated and have wide acceptability amongst clinical and patient groups. The use of validated PRO tools will improve production of high-quality data from the registry. By having clear policies on data governance and management that are acceptable to all its stakeholders including patients, health care professionals, researchers and industry, EuRRECa aims to sustain itself beyond the current lifetime of the project.

### 3.1. Platform for e-Reporting of Rare Conditions (e-REC)

e-REC (https://eurreca.net/e-rec/), launched in 2018, is an electronic reporting system, which allows monthly reporting of new clinical cases by participating centres. It also allows clinical networks such as Endo-ERN and ERN-BOND to objectively map the activity within the network. In the current platform, new users can select the conditions and age groups (<18 or ≥18 years) that they want to report on, with the option to report suspected and/or confirmed cases. This is particularly useful in cases where genetic or biochemical diagnostic confirmation is pending. Multiple reporters can be selected within each reporting centre; however, it is not possible for another team member to sign up to report on the same condition within the same age group. Unique IDs for reported cases are generated instantaneously and emailed to users to be stored locally at reporting centres. The reported data do not contain any personally identifiable information and patient consent is not required to report cases through the e-REC platform. In addition, public information sheets are available in several languages (https://eurreca.net/e-rec-information-sheets-2/). Data are stored on a secure server in the University of Glasgow and are available to all stakeholders following approval by the Data Access Committee of the EuRRECa project. Since the launch of the e-REC platform, a total of 60 centres from 26 countries have registered to participate in e-REC with over 5500 cases reported to date. Further details of the contents of this registry are available in the progress reports that are available at https://eurreca.net/e-rec-reports/.

The e-REC data collection system is designed to minimise the reporting burden on health care professionals and its purposes include: (1) understanding the level of activity that is occurring amongst the participating centres, (2) provision of data to participating European Reference Networks such as Endo-ERN and ERN-BOND for their continuous monitoring exercise, (3) understanding how many people are affected by a particular rare condition, (4) providing feasibility data for future research studies and (5) understanding the process of diagnosis and initial presentation of rare conditions through secondary surveys.

### 3.2. Core Registry

The EuRRECa Core Registry (https://eurreca.net/core-registry/), operational since June 2019, collects a core dataset created using existing standards [5] that ensure that the fields have a high level of interoperability for a wide range of conditions including those that are covered within Endo-ERN and ERN-BOND. By using core data elements that have been agreed at a very wide level across Europe, the Core Registry allows interoperability across several rare disease registries. Clinicians enter patient data into the Core Registry after obtaining consent from patients. Reference centres within Endo-ERN can use the opt-in consent form that is provided by the ERN; however, more detailed information sheets and opt-in consent forms that have ethics and information governance approval in the UK have also been created specifically for the EuRRECa Core Registry and are available in several languages (https://eurreca.net/core-registry-information-sheets/). Irrespective of the nature of the consent process, participating centres and clinicians are advised to seek local information governance and ethics approval. The ethics approval for the Core Registry also allows the exchange of data in the Core Registry and other EuRRECa-approved registries. Patients can also access the Core Registry to view their own record, set preferences for data sharing, request deletion of their data, receive newsletters and complete patient reported outcomes (PROs). The focus on patients in the Core Registry also opens up the opportunity to provide more customised information on their condition as well as studies. The Core Registry incorporates generic and condition-specific, clinician and patient reported markers of outcomes and will develop a list of affiliate disease registries for effective signposting and data sharing. All data that are collected in the registry are accessible to researchers following approval by the Data Access Committee. Since the launch of the Core Registry in June 2019, there are currently almost 400 cases that have been entered from 10 centres in nine countries in Europe. Further details of the contents of this registry are available in the progress reports that are available at https://eurreca.net/reports/.

The Core Registry will be used to collect data that are collected during routine clinical care. It is anticipated that, in time, these data will be used for several secondary purposes by a wide range of stakeholders to: (1) provide a source population for the conduct of clinical trials, (2) provide the patient with details of their condition, (3) provide information on specific interventions related to defined patient groups, (4) provide for the follow-up of small patient populations, (5) provide life-cycle assessment of the effectiveness and safety of interventions and medicinal products, (6) provide robust data on disease epidemiology, including the distribution, patterns and presentation of disease conditions in defined populations, patients’ characteristics and current standard of care and (7) provide source population data that can be linked to other datasets on specific outcomes.

## 4. Data Access

### 4.1. EuRRECa Data Access Committee

Sharing deidentified individual-level health research data is widely promoted and has many potential benefits. However, there are also some potential harms, such as misuse of data and breach of participant confidentiality. One way to promote the benefits of sharing while ameliorating its potential harms is through the adoption of a managed access approach where data requests are channelled through a Data Access Committee (DAC), rather than making data openly available without restrictions [12].

Within the EuRRECa project, the DAC consists of a Core DAC including the Chair and Co-Chair and the project management team. In addition, there is representation from partner projects such as EuRR-Bone, allied European Reference Networks including Endo-ERN and ERN-BOND, professional scientific societies such as ESPE, ESE and ECTS (European Calcified Tissue Society) and patients. The DAC also relies on a group of experts who are co-opted depending on the project theme of the submitted application. The main roles of the DAC include: (1) checking that the proposed work complies with the terms and conditions of the ethics approval provided to the EuRRECa project, (2) looking for evidence of the third party who is requesting that the data are appropriately qualified for use of the data, (3) advising on improving the project and any overlaps with ongoing projects, (4) advising on the dissemination and publication plan, (5) ensuring that the effort of all those involved is appropriately acknowledged, (6) responding to all data requests promptly, (7) communicating to the requestor with appropriate feedback, (8) being aware of their own conflicts of interest, (9) treating all data requests confidentially and (10) reviewing and advising on the governance processes within the DAC. The overall aim of the DAC is to promote the research use of the data that are being collected in the EuRRECa registries through a transparent and simple approach whilst ensuring the long-term sustainability of the EuRRECa project.

### 4.2. Data Access Policy

The EuRRECa data access policy (DAP) applies to all data requested from both registries (e-REC and Core Registry) and was developed by EuRRECa Work Packages 1 (Management and Coordination), 2 (Dissemination and Access) and 6 (Registry and Reporting). Data may be requested for a number of reasons that may include conducting an epidemiological survey for a particular condition, designing a study or survey, preparing a case for funding, appraising best clinical practice or comparing outcome measures across different conditions or centres.

### 4.3. Stakeholders Accessing Data

The broad groups of stakeholders that will require access to data within e-REC include e-REC reporters, e-REC analysts, external researchers and the project management team, with the latter group having access to all data and the capacity to provide role based access. Reporters have access to the data of cases reported on a monthly basis in their centre, whilst analysts have read-only access to this data. External researchers will not have access to any data and are required to complete a data request form and data sharing agreement to obtain data.

The broad groups of stakeholders that will require access to data within the Core Registry include patients, clinicians providing health care, centre administrators, centre leads, researchers and the project management team. Patients have read-only access to their own data and are able to complete patient reported outcome questionnaires online. Centre leads are responsible for governance at their local centre and are able to provide access to other members of their clinical team. Clinicians also have access to all the cases at their centres.

### 4.4. Data Ownership

The University of Glasgow is the owner and sponsor of the EuRRECa registries project. In the Core Registry, the patient participant (who is the “data subject”) and/or the legal guardians, in case the patient participant is under the age of 16, is/are the primary owner(s) of the data and will grant each of the users and the University of Glasgow a nonexclusive licence to use such data for research purposes. The institution of the clinician who has entered the data is the owner of the aggregated data of that patient participant and acts as the “data controller” at the local centre. The principal investigator of the EuRRECa project is the custodian of the data and acts as the “data controller” of the registries and also as the “data processor” with responsibility for the protection of the data, its storage, use and access. When processed, the data become research data and are then the intellectual property of the investigator who is the “third party”. Moreover, this third party has to abide by the agreement reached in the data sharing agreement whilst using the data supplied for the purpose stated in the data request form.

### 4.5. Ethics

The Core Registry (https://eurreca.net/ethics-approval-coreregistry/) and e-REC (https://eurreca.net/ethics-approval-erec/) have been approved in the UK by the West of Scotland Research Ethics Committee as “research databases” and the data governance standards in the e-REC and the Core Registry comply with GDPR. Furthermore, a research project using data from registries will be considered to have ethics approval subject to the following conditions: (1) the research project is within the fields of research described in the application, (2) the research protocol has been subject to scientific critique by the Data Access Committee, (3) the processing of the data in the Core Registry will comply with the terms of informed consent from data subjects and (4) research must be conducted in circumstances such that data subjects are not identifiable to the external researchers. Data must be effectively anonymised or pseudonymised prior to release to external researchers; the researchers should treat datasets in confidence and not attempt re-identification of data subjects through linkage with other datasets. (5) A data sharing agreement must be in place with all external researchers to ensure processing of the data in accordance with the terms of the ethics approval and any other conditions required by the project management team. Any research project that requires external researchers to be able to identify data subjects for linkage with other datasets, for collecting further data from subjects or for undertaking other research procedures involving subjects, is not covered by this approval. Such projects should be the subject of further project-specific applications for ethics reviews. All centres who consider participating in the two registries (e-REC and Core Registry) should consider themselves as local data controllers and secure approval from local institutions before contributing data. On approval of any research project by the DAC, approval from individual centres will be obtained before their data are shared for any research project. Whilst ethics approval already exists to use the data for research under the conditions mentioned above, local regulations may vary and individual centres may also want to check whether they need any additional approvals locally.

### 4.6. Procedures for Obtaining Data

**Figure 2:**
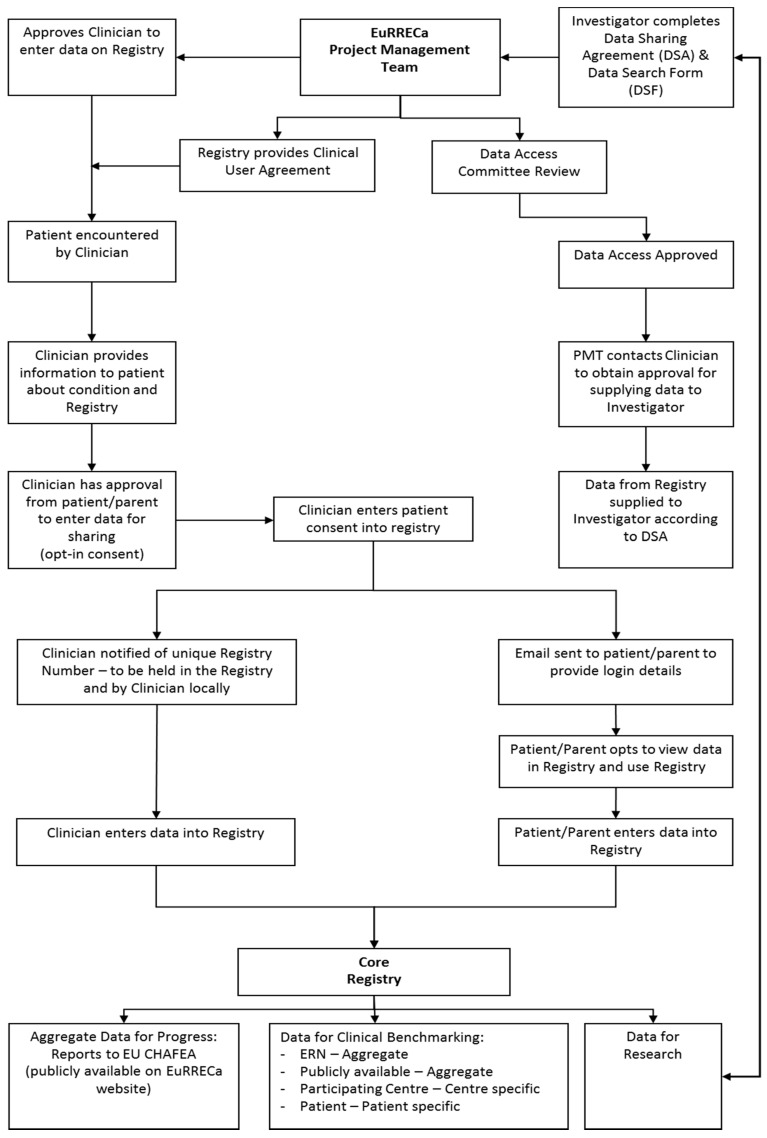
Data Flow within the European Registries for Rare Endocrine Conditions (EuRRECa) Core Registry.

**Figure 3:**
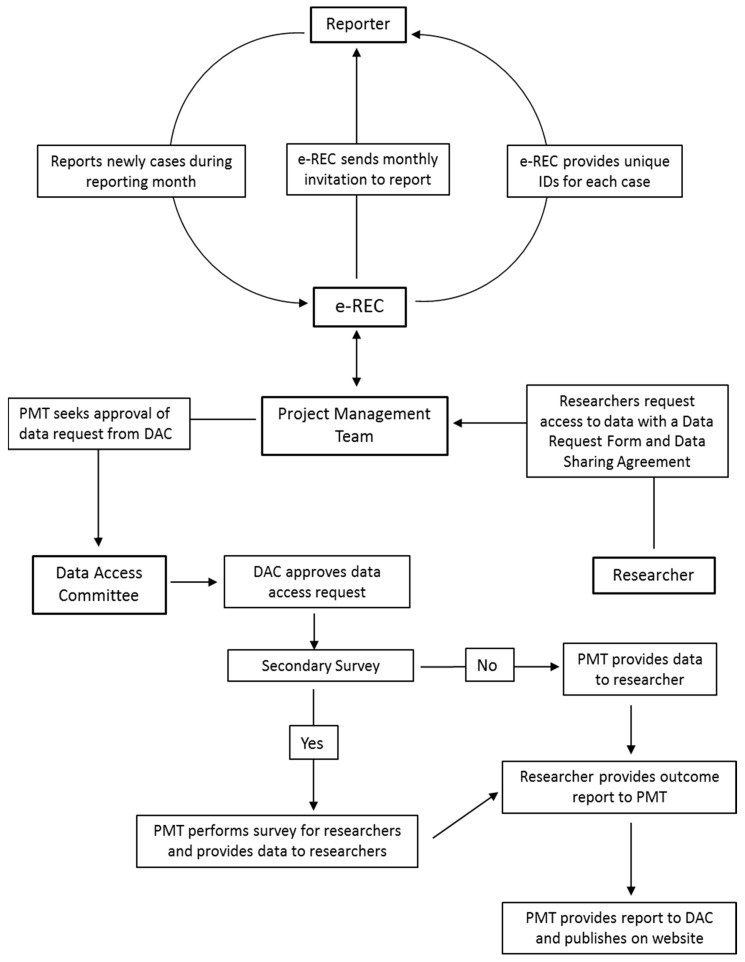
Data flow within the e-Reporting of Rare Conditions (e-REC) platform.

**Figure 4:**
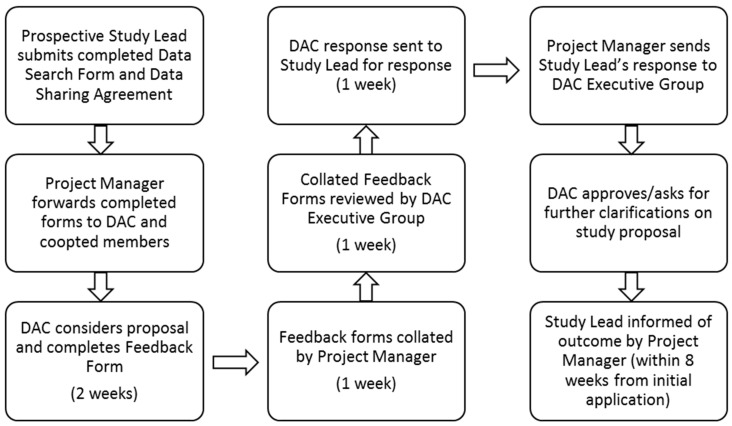
EuRRECa data access committee approval process.

Data flow within the Core Registry and e-REC is outlined in Figure 2 and Figure 3 and the process for obtaining data for research is managed by a project manager. All prospective investigators are required to contact the EuRRECa project management team who can also provide extensive support to the investigators and guide them through the application process. The support provided may include advice on the proposed study design and liaising with the centres that use the Core Registry and e-REC on behalf of the study investigators. Prospective investigators will also be provided with appropriate guidance for completing the study documents that are sent to the Data Access Committee for study approval. To obtain access to data, the investigator is required to complete a data request form and a data sharing agreement. The request is reviewed by the DAC who provide feedback to the applicant, who may be asked to revise the request. Once approved, data are released to the applicant. The process for seeking access is summarised in Figure 4. In some cases, further information may be required and via the project management team, the DAC can ask for additional data or clarification of data they have already received. In case the contents of a new application overlap with an existing active application, the investigators of the two applications will be jointly advised to discuss the overlap and in all cases, supply of data is dependent on feedback from the recipients in the form of a report which is posted on the public area of the EuRRECa website.

### 4.7. Quality Assurance

The data in e-REC and the Core Registry shall undergo a 6-monthly analysis by the project management group to provide progress reports to the DAC. This analysis will focus on overall data accrual, content, quality and headline descriptions of care. This analysis will not require approval from the DAC but shall be performed closely with the oversight of the DAC. It is anticipated that the regular use of the data for research will also lead to an improvement in the quality of the data held in the registry. In addition, EuRRECa has a work package that oversees quality assurance. This work package was instrumental in ensuring the data being collected in the Core Registry adhere to the FAIR principles. By using internationally recognised diagnostic classifications and universal standards such as Orphacodes and logical observation identifiers names and codes (loinc.org), the data shall be highly interoperable with other registries that use the same approach.

### 4.8. Sustainability

Although the EuRRECa project is in its infancy, its sustainability will depend on the opportunities that present themselves as the project develops. A pan-European surveillance system as well as a core registry are novel projects that have the potential to deliver many benefits to endocrinology in the long term. The platforms are an exemplar not just for endocrinology across the world but also for other rare conditions. By offering a “menu” of registries including the e-REC, the Core Registry and a list of vetted registries that have an affiliate status, EuRRECa caters for all health care providers who may have a variable extent of resources for using registries and it is anticipated it will become the gateway to participation in RD registries. By ensuring that all the resources that are developed by EuRRECa are in the public domain, the project will ensure that good standards of data collection and governance will become routine practice in endocrinology. The e-REC platform has the ability to act as a tool for project and clinical trial feasibility assessments and has already started displaying its utility as a global surveillance project together with ESE of Covid-19 endocrine cases [13]. Thus, not only does e-REC act as a tool for continuous monitoring of reference centres within ERNs, it can form the basis for performing several audits and practice reviews. By collecting commonly agreed core outcomes in the Core Registry, it is anticipated that EuRRECa will facilitate the development of benchmarks of care which can be used for improvement in care quality within the ERNs and other reference centres that want to participate.

## 5. Conclusions

With the recent expansion of RD registries, it has become even more essential to highlight vital aspects of data access and data governance policies for RD registries. In this article, we discussed the European Registries for Rare Conditions (EuRRECa) which has incorporated the development of a Core Registry and an e-Reporting Programme for Rare Conditions. EuRRECa adheres to the highest standards of data security and information governance and is an example of a project that aims to promote good standards of practice for improving the quality of utilisation of RD registries. Now that the processes on data governance have been developed, the next step is to encourage data collection and data provision for research. Mechanisms that allow the use of machine learning technology within the bounds of good data governance will also require exploration [14]. These activities will be critical for the long-term sustainability of EuRRECa. The lessons learnt from this model can be applied to many other groups of rare conditions that require a common Core Registry and an electronic surveillance system to capture the activity, epidemiology and natural history of these conditions. This framework can also be used for improving the governance structure of new and existing registries for rare conditions.
